# The denture microbiome in health and disease: an exploration of a unique community

**DOI:** 10.1111/lam.13751

**Published:** 2022-06-09

**Authors:** J. Redfern, L. Tosheva, S. Malic, M. Butcher, G. Ramage, J. Verran

**Affiliations:** ^1^ Department of Natural Sciences, Faculty of Science and Engineering Manchester Metropolitan University Manchester UK; ^2^ Department of Life Sciences, Faculty of Science and Engineering Manchester Metropolitan University Manchester UK; ^3^ Department of Oral Sciences, Glasgow Dental School, School of Medicine, Dentistry and Nursing University of Glasgow Glasgow UK

**Keywords:** antimicrobials, biocontrol, biofilms, diseases, diversity

## Abstract

The United Nations suggests the global population of denture wearers (an artificial device that acts as a replacement for teeth) is likely to rise significantly by the year 2050. Dentures become colonized by microbial biofilms, the composition of which is influenced by complex factors such as patient’s age and health, and the nature of the denture material. Since colonization (and subsequent biofilm formation) by some micro‐organisms can significantly impact the health of the denture wearer, the study of denture microbiology has long been of interest to researchers. The specific local and systemic health risks of denture plaque are different from those of dental plaque, particularly with respect to the presence of the opportunist pathogen *Candida albicans* and various other nonoral opportunists. Here, we reflect on advancements in our understanding of the relationship between micro‐organisms, dentures, and the host, and highlight how our growing knowledge of the microbiome, biofilms, and novel antimicrobial technologies may better inform diagnosis, treatment, and prevention of denture‐associated infections, thereby enhancing the quality and longevity of denture wearers.

## Graphical abstract

Dentures are becoming increasingly common. Since colonisation (and subsequence biofilm formation) by some microorganisms to dentures can significantly impact, the study of denture microbiology has long been of interest to researchers.

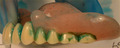

## Introduction

A denture is an artificial device designed to act as a replacement for one, multiple (partial dentures) or all (full dentures) teeth. Despite improvements in oral health, the need for full or partial dentures is expected to increase as the global population over the age of 65 years is expected to double by 2050 (United Nations 2019). The older population is also likely to have general and multiple health complications that make treatment planning around oral health more difficult (PHE 2015). Although the number of denture wearers globally is very high, with almost 41 million users in the United States alone in 2020 (Statista 2020), evidence of an understanding relating to health implications of long‐term denture use is relatively sparse in the literature. For example, one study of 67 denture wearers reported that almost 40% of patients ceased to wear their dentures within 5 years due to a variety of reasons, including pain, discolouration, and difficulty of use (Koyama *et al*. 2010). While it is clear that denture‐related disease is a multifactorial phenomenon, undoubtedly oral health is predicated in the status of the oral microbiome. Understanding how microbes relate to denture wearing has the capacity to improve oral and systemic health of the global elderly population.

**Figure 1:**
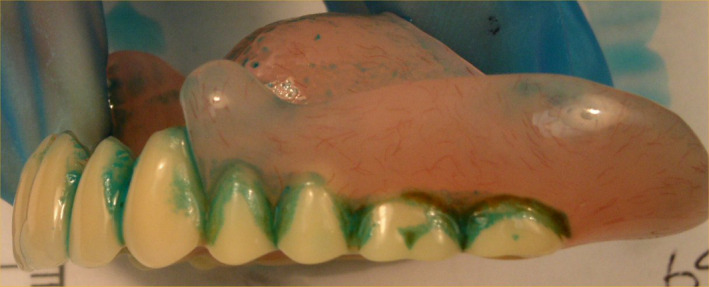
Example of a denture stained with plaque disclosure (blue), showing plaque accumulation on the upper fitting surface of the denture, and between the teeth of the prosthesis. [Colour figure can be viewed at wileyonlinelibrary.com]

As dentures are not sterile and are used at the body‐external environment interface, it is possible to be colonized by micro‐organisms (Olms *et al*. 2018) (Fig. 1). There are complex and numerous interactions between the individual (age, health), their denture (age, material, hygiene/cleaning regime) and colonizing micro‐organisms (nature of microbiome/biofilm, and potential infection risks), which will be considered in this review. The oral environment differs between the dentate and edentate mouth: the tooth is replaced by an inert removable prosthesis; the fitting surface of the denture provides a unique protected environment; the gumline is absent in complete denture wearers, and the natural dentition that abuts a partial denture is particularly prone to caries and gum disease (Zlatarić *et al*. 2002). The microbiology in these different scenarios is varied, and should be considered separately, particularly for partial dentures and implants, where the many different surfaces, interfaces, and locations will likely all bring additional complexities. This review focuses particularly on the complete denture (fitting and external surfaces).

## Micro‐organisms associated with dentures

As with natural dentition, a denture surface once placed in the mouth becomes coated with an ‘acquired pellicle’ of salivary glycoproteins (including salivary amylase, albumin, mucin, and lysozyme) and immunoglobulins (Edgerton and Levine 1992; Marsh *et al*. 2016; Chawhuaveang *et al*. 2021). One study on polymethylmethacrylate (PMMA), the most commonly used material in dentures) reported that acquired pellicles primarily consisted of lyzozyme and histatins, in comparison to those forming on dentine that consisted of carbonic anhydrase, carbonate dehydrastase, cystatins and lyzozyme, and histatins (Svendsen and Lindh 2009). This coating of salivary product provides adhesion receptors facilitating the adhesion and colonization of micro‐organisms (Edgerton *et al*. 1993; Mukai *et al*. 2020). The profile of the acquired pellicle on the enamel (and therefore likely denture) is not consistent, and can change depending on the location of the dental arch (Ventura *et al*. 2017). Another study reported that microbial colonization is not necessarily determined by denture material, with both PMMA and polyacrylamide supporting biofilm growth (Olms *et al*. 2018). However, the pellicle composition might vary, and influence the identity of primary colonizers, though initial colonization may not necessarily differ between a range of dental materials (Mukai *et al*. 2020).

In the oral cavity, primary colonizers of hard/enamel surfaces include Gram‐positive *Streptococcus* spp. (*S. oralis*, *S. mutans*, *S. mitis*, *S. gordonii*, *S. sanguinis*, and *S. parasanguinis*), and other species including *Veillonella* spp., *Neisseria* spp., *Rothia* spp., *Abiotrophia* spp., *Gamella* spp. and *Granullicatella* spp. (previously belonging to the nutritionally variant streptococci) (Theilade *et al*. 1983; Aas *et al*. 2005; Yitzhaki *et al*. 2018). Secondary colonizers can adhere to and coaggregate with primary colonizing micro‐organisms, which if adhered to a denture can form complex denture plaque communities (Coulthwaite and Verran 2007; Jenkinson 2011). More specifically, studies on the cultivable flora of denture plaque have focused on facultative anaerobes on the denture‐fitting surface. However, as with dental plaque, obligate anaerobes are also reported in more mature plaque, and may be used as an indicator of plaque maturity—and thence poor denture hygiene (Coulthwaite *et al*. 2005). Despite the prevalence of bacteria in denture plaque, the most commonly studied micro‐organism is the yeast *Candida albicans* (Verran 1998; Ramage *et al*. 2004; Gleiznys *et al*. 2015), and to a lesser extent other *Candida* species such as *C. glabrata* (Coco *et al*. 2008; Zomorodian *et al*. 2011), *C. famata*, *C. dubliniensis*, and *C. tropicalis* (Zomorodian *et al*. 2011; Gauch *et al*. 2018). Notably, to date, there have been no confirmed reports of pan‐antifungal resistant yeast *Candida auris* isolated from dentures, though its continued global spread makes this a matter of ‘when’ and not ‘if’. Indeed, it can be found readily in the anterior nares of patients (Malczynski *et al*. 2020), which means *C. auris* is able to readily colonize dentures. Moreover, in critically ill patients, it has already been shown in outbreaks that it is possible to isolate it from oral samples (Biswal *et al*. 2017).

The interest in *Candida* in denture plaque derives from its association with denture stomatitis, a term which describes inflammation of the epithelial surfaces in contact with the denture, particularly the maxillary denture (Salerno *et al*. 2011). *Candida* spp. are well‐known secondary colonizers of denture plaque, with data suggesting *C. albicans* can co‐aggregate with *Streptococcus* spp. and result in biofilm on saliva‐coated surfaces (Bamford *et al*. 2009). The yeast is found primarily on the fitting surface of the maxillary denture. The enclosed environment, the presence of pre‐existing plaque, the protective nature of the surface topography and the acidogenic nature of the plaque have all been proposed as factors which enhance survival (Verran 1988). It has also been demonstrated recently that *C. albicans* acts as a ‘keystone’ commensal (given its relationship to coexist alongside human bacterial pathogens) within relevant oral biofilm model systems, with the suggestion that the larger physical nature of this dimorphic yeast makes it capable of creating physical and chemical microenvironments that support smaller bacteria, and obligate anaerobes resulting in increased biomass and metabolic activity (Janus *et al*. 2016; Young *et al*. 2020).

Although publications focusing on denture plaque are far fewer than those about dental plaque, there have been some seminal studies in the area, stemming from the 1980s. Theilade and Budtz‐Jørgensen (1988), examined the cultivable flora from the fitting surface of dentures of eight patients. Findings revealed *Streptococcus* spp. (in particular *S. mutans, S. mitis, S. salivarius, and S. sanguis*) dominated, persisting in 17–76% of samples. Gram‐positive rods *Actinomyces* spp. (*A. israelii, A. naeslundii, A. odontolyticus*) and lactobacilli, and *Veillonella* spp. were also present. Gram‐negative rods and yeasts were identified in smaller amounts. These findings are also supported by other studies (Budtz‐Jørgensen 1981; Walter and Frank 1985; Lamfon *et al*. 2005). *Staphylococcus* spp. and *Micrococcus* spp., commonly found on skin and in the environment are rarely found in the oral cavity but have been isolated from denture plaque taken from clinical cases of denture stomatitis (Kulak *et al*. 1997; Webb *et al*. 1998). Essentially, culture studies of denture plaque have shown that it is a diverse microbial biofilm, structurally similar to dental plaque (Budtz‐Jørgensen 1981; Walter and Frank 1985), with a similar microbial composition (Nikawa *et al*. 1998), but with elevated levels of yeasts (primarily *Candida* spp.), *Lactobacillus* spp., streptococci and staphylococci (Theilade and Budtz‐Jørgensen 1988; Marsh *et al*. 1992). These elevated levels have been shown to be particularly notable in cases of denture stomatitis (Theilade and Budtz‐Jørgensen 1988), and have been found to increase with the increase in age of the denture (Budtz Jorgensen 1974; Theilade *et al*. 1983; Mizugai *et al*. 2007). It is not surprising that the overall microbial composition of denture plaque is similar to that of dental plaque, since the underlying oral environment provides similar conditions. However, as noted earlier, on the inert denture, there is less intimate contact with body fluids (serum, blood), and more occluded spaces (the fitting surfaces) with less movement of saliva and more retention of food, which might encourage the presence of less fastidious (and even physically larger) species. Although *Candida* spp. are of particular concern in denture wearers due to their strong association with denture stomatitis, their reported proportion in denture plaque in comparison to bacterial isolates is relatively low (Theilade and Budtz‐Jørgensen 1988). Indeed, denture stomatitis has been associated with ‘dirty’ dentures and poor denture hygiene, as well as with the presence of *C. albicans* (Webb *et al*. 1998).

Whilst such investigations have been able to inform on micro‐organisms associated with dentures, classic microbiological techniques are not able to culture all micro‐organisms onto agar. First described by Staley and Konopka (1985), the ‘Great Plate Count Anomaly’ describes the phenomenon that the majority of micro‐organisms are nonculturable on agar, limiting the ability to discover the true microbial community of an environment—particularly one so complex as plaque—if only reliant on culture‐based techniques.

## Micro‐organisms associated with dentures: nonculture‐based assessments

As described above, the denture microbiota is composed of a wide range of both eukaryotic and prokaryotic micro‐organisms, but not all are culturable. Therefore, modern molecular biology techniques that do not rely on culturing have gained popularity in the study of denture‐related plaque. Amplicon sequencing is one such technique, whereby specific genes (16S, 18S and ITS for bacterial, fungal, and microbial eukaryotes respectively), which are present in all but unique for each species, are sequenced and analysed using computer‐based bioinformatics. Another method, quantitative polymerase chain reaction (qPCR), analyses the amount of a specific gene (usually the 16S rRNA gene for bacteria, or 18S, or internal transcribed region [ITS] for fungi) in a sample without the need to sequence. As the genetic material needed for such analysis can be extracted directly from a sample, it removes the requirement to culture micro‐organisms, and is therefore more likely to provide a more representative picture of the complex communities found on dentures. Using molecular biology techniques, it is estimated that the oral microbiome (the term commonly used to describe the whole community of micro‐organisms in an environment) contains over 700 species of bacteria (Verma *et al*. 2018; Deo and Deshmukh 2019). Whilst such studies specifically looking at dentures are limited compared to the wider oral cavity, they nevertheless provide a more accurate picture of the microbial population of a denture, and how this might be associated with other factors such as disease state.

To date, there is a limited collection of studies that have embraced 16S‐based sequencing approaches to better understand the microbial complexity upon dentures (Table 1). Campos *et al*. (2008) was the first to use a prenext‐generation sequencing approach (16S rDNA cloning) (Campos *et al*. 2008). Here, over 82 different bacterial species were identified, of which 29 were exclusive to disease and 26 to healthy denture wearers alongside *Candida* sp. in both. The first microbiome study was performed by O'Donnell *et al*. (2015), where it was shown that bacteria from the taxa Bacilli and Actinobacteria were most abundant on the mucosa and denture of 130 patients, with *Lactobacillus* spp. showing a positive correlation in those with higher quantities of *Candida* spp. Despite no significant difference in overall microbiota between the dentures of healthy vs inflamed mouths, a followup study by these authors using qPCR found that dentures could be a reservoir for respiratory pathogens, including *Streptococcus pneumoniae*, *Pseudomonas aeruginosa*, *Haemophilus influenzae B*, *Streptococcus pyogenes*, and *Moraxella catarrhalis* (O'Donnell *et al*. 2016). In a smaller study, Shi *et al*. (2016) reported the genus *Actinomyces* was most pervasive on both dentures and remaining teeth, followed by *Streptococcus*, *Veillonella*, *Capnocytophaga*, *Neisseria*, *Prevotella*, and *Corynebacterium*, independent of the surface or health status, while the microbiome of dentures from stomatitis patients was more diverse than dentures belonging to healthy patients (O'Donnell *et al*. 2016). Additional data (Fujinami *et al*. 2021) revealed that when comparing 30 denture samples with 16 plaque samples, *Streptococcus* sp., *Lactobacillus* sp., and *Corynebacterium* sp. were more abundant in denture plaque than in dental plaque likely due to the aerobic conditions on the denture surface as the denture is often removed from the mouth or in contact with saliva. Microbial association with malodour has also been studied, with Yitzhaki *et al*. (2018) using 16S sequencing to conclude the phyla *Firmicutes* and *Fusobacteria* and the genera *Leptotrichia*, *Atopobium*, *Megasphaera*, *Oribacterium*, and *Campylobacter* alongside a generally more diverse and significantly different microbial population were associated with denture malodour compared to samples from nonmalodour patients (Yitzhaki *et al*. 2018). It should be noted that the study design, DNA extraction protocols, storage, sequencing platform, and primers are not consistent from one study to the next, so absolute comparison is not possible (Table 1). However, these studies have started to provide a greater insight into the diversity of bacteria that occupy these substrates.

**Table 1:** Summary of published denture microbiome studies and their key characteristics

Study	Location	Design	Controls (no. patient’s)	Cases (no. patient’s)	Extraction method	Storage	Sequencer	Region	Accession	Key findings
Campos *et al*. (2008)	São Paulo, Brazil	Cross‐Sectional	Healthy (10)	Denture stomatitis(10)	QuickExtract	NA	ABI PRISM 3100 Genetic Analyzer	16S	AY672070‐76	Distinct differences in microbiome between health and disease
O'Donnell *et al*. (2015) and Delaney *et al*. (2019)*	Glasgow, UK	Cross‐Sectional	Mucosa/plaque	Denture (130)	AGOWA mag Mini DNA Isolation Kit	−80°C	MiSeq	16S V4, ITS1	PRJNA324548	Distinct associations between Candida load, oral hygiene and microbiome
Shi *et al*. (2016)	Los Angeles, USA	Cross‐Sectional	No denture stomatitis (10)	Denture stomatitis (10)	DNeasy Blood and Tissue kit	NA	454	16S V1‐V3	PRJNA292354	Denture microbiome is reflective of that of teeth but identifiably distinct when comparing health and disease
Asakawa *et al*. (2018)	Fukuoka, Japan	Cross‐Sectional	Tongue (506)	Denture (137)	IsoQuick	−80°C	Ion PGM	16S	DRA006979	Diminished oral health and hygiene is reflected in the microbiota of the tongue
Yitzhaki *et al*. (2018)	Ramat‐Aviv, Israel	Cross‐Sectional	NA	Denture (26)	Genomic DNA Mini Kit	−20°C	MiSeq	16S V3‐V4	On request	Microbiome analysis suggests population distinction between olfactorily distinct samples
Nedumgottil (2018)	Puducherry, India	Cross‐Sectional	NA	Denture (88)	NucleoSpin Microbial DNA mini kit	NA	NA	16S	On request	Denture wearers linked to co‐occurrence of *Streptococcus mutans, Veillonella atypica*, and *Granulicatella adiacens*
Morse *et al*. (2019)	Cardiff, UK	Cross‐Sectional	No denture stomatitis (11)	Denture stomatitis (8)	Gentra PureGene Bact/Yeast DNA extraction kit	−20°C	MiSeq	16S V1‐V3	On request	Reduced bacterial diversity may lead to dysbiosis in DS
Mukai *et al*. (2020)	Yokohama, Japan	Cross‐Sectional	Saliva (8)	Denture (8)	ISOSPINE Fecal DNA Kit	−80°C	MiSeq	16S V3‐V4	PRJNA592277	Microbial diversity diminished on denture surface compared to saliva
Murugesan *et al*. (2020)	Doha, Qatar	Cross‐sectional	Healthy (861)	Denture (136)	QIAsymphony	−80°C	MiSeq	16S V1‐V3	On request	*Streptococcus* and *Neisseria* found to be more abundant in denture wearers
Fujinami *et al*. (2021)	Nagoya, Japan	Cross‐Sectional	Plaque (16)	Denture (30)	MasterPure DNA Purification Kit	−20°C	MiSeq	16S V3‐V4	DRA011478	Pathogens associated with aspiration pneumonia were more commonly isolated from dentures
Grischke *et al*. (2021)	Hannover, Germany	Cross‐Sectional	Healthy (372)	Peri‐implantitis/denture (725)	QIAshredder Mini Spin	−80°C	HiSeq	ND	PRJEB43417	Removable dentures are identifiable as a risk‐factor for peri‐implantitis

* Two studies use same dataset for different analysis question.

As reported using culture techniques, *Candida* is present as part of the denture microbiome (Campos *et al*. 2008). In a study analysing 82 Dutch denture wearers, high *Candida* loads were associated with the bacterial class Bacilli, negatively associated with bacterial classes Fusobacteria, Flavobacteria, and Bacteroidia, and were generally less diverse and dominated by streptococci (Kraneveld *et al*. 2012). Whilst such studies are useful and provide data on the potential relationship between denture stomatitis, denture plaque, and the denture microbiome, the literature is limited, lacking many large study populations/sample sizes, and making generalization difficult. To date, there are only 12 independent studies available in the public domain that have assessed the denture plaque microbiome, and which have involved the analysis of over 1000 patients’ dentures (Table 1). Among the limited availability of research, there are related concerns for the lack of unanimity in methodologies presented. It has been evidenced in previous work, for example, microbiome profiling conducted by Teng *et al*. (2018), that extraction protocols can significantly impact sample‐to‐sample diversity. Moreover, community diversity has also been associated with the specific 16S region selected (Bukin *et al*. 2019; Chen *et al*. 2019) which has been shown to vary from study to study (Table 1). However, it is likely there will be an expansion and increase in uniformity of these over the next few years as attempts are made to use microbiome studies as a diagnostic tool.

## Dentures and biofilm: denture plaque

Physiologically, a harmonious relationship exists between the oral microbiota and the host. Oral micro‐organisms naturally thrive in areas where salivary flow is low, such as areas between the teeth and gingival crevices, in addition to the fitting surface of a denture. Micro‐organisms are found in complex communities on the denture (Fujinami *et al*. 2021), attached to the denture surface or to other cells whilst embedded in extracellular polymeric substance (Gendreau and Loewy 2011). It is widely agreed in the literature that micro‐organisms displaying this phenotype, and known as biofilm, present increased resistance to antimicrobial treatments whilst also being physically difficult to remove from the substratum (Sharma *et al*. 2019). There have been many reviews published on the formation of biofilms generally and dental plaque biofilms specifically (e.g. Subramani *et al*. 2009), with fewer concerning denture plaque (Hannah *et al*. 2017). Although the phenomenon of biofilm formation is similar for denture and dental plaque, the particular environment between the denture and the roof of the mouth has been shown to be microbially distinct (O'Donnell *et al*. 2015). The presence of *C. albicans* at this site has resulted in significant attention paid to factors affecting its colonization of denture surfaces (Ramage *et al*. 2004; Pereira‐Cenci *et al*. 2008), with a view to prevention or control (Rautemaa and Ramage 2011).

As interest in denture stomatitis increased during the 1980s, studies on the attachment of *C. albicans* to denture PMMA and silicone began (Samaranayake and MacFarlane 1980; Pereira‐Cenci *et al*. 2008; Rodger *et al*. 2010). The yeast readily attached to the surface, with retention being enhanced by increased surface roughness (Verran and Maryan 1997; Jackson *et al*. 2014; Verran *et al*. 2014), lowered pH (Verran *et al*. 1991), and other factors. The introduction of salivary pellicle and other conditioning films into this simple system (Nikawa *et al*. 1992), the ability of *C. albicans* to exist in a yeast and hyphal form (Jackson *et al*. 2014), and the presence of primary colonizers (Verran and Motteram 1987), introduced complexity in a step‐wise manner and provided additional information. *In vitro* studies on the interactions occurring between *C. albicans* and *Streptococcus mutans* are common (e.g. Baena‐Monroy *et al*. 2005; Falsetta *et al*. 2014; Zhou *et al*. 2018). Initially, this might seem counter‐intuitive, because *S. mutans* is best known for its role in dental caries (Hamada *et al*. 1984), and it may not be the most common streptococcus present in denture plaque, with *S. sanguinis* historically recovered more often (Carlsson *et al*. 1969). However, the highly acidogenic and aciduric nature of the environment generated by *S. mutans* would likely prove beneficial for yeast proliferation. These basic studies exploring coaggregation and antagonistic behaviours have fuelled an explosion of studies that specifically explore interkingdom interactions (Delaney *et al*. 2018, 2019).

Materials used in conjunction with the denture were also assessed for their susceptibility to colonization. For example, penetration of denture soft‐liner, used to make the denture more comfortable for the wearer, has been demonstrated (Bulad *et al*. 2004; Rodger *et al*. 2010; Todd *et al*. 2019). The search for novel materials that might be less conducive to *Candida* colonization, but which retained the physicochemical properties required for their *in vivo* implementation, is ongoing and will be considered later.

More complex studies on denture plaque models have been carried out, for example, using a denture plaque microcosm (Coulthwaite and Verran 2007, 2008; Brown *et al*. 2022), and biofilm models, used to assess the impact of putative antimicrobial/antibiofilm agents (Sherry *et al*. 2016; Brown *et al*. 2022). In line with Marsh’s ‘Ecological Plaque Hypothesis’ (Marsh 1994), it has been proposed that denture stomatitis arises from a shift in the plaque microbiology away from health due to external changes such as increased plaque acidogenicity and increased plaque quantity (Verran 1998)—and perhaps diversity (Marsh 1994). Thus, both control of plaque quantity and management of specific aspects of the microcosm might enable progress towards maintenance of a healthy denture plaque.

## Health and ill‐health associated with dentures

Through the use of *in vivo* cultural studies and *in vitro* denture models, knowledge of denture plaque microbiology is now substantial. Studies on healthy and diseased denture plaque are hampered by plaque complexity, as well as the relatively small research funding available to investigators in comparison to that for the study of dental plaque. Nevertheless, there are several health‐related problems associated with denture plaque (Coulthwaite and Verran 2007). As has been noted, denture plaque is a key aetiological factor in the oral mucosal inflammatory disorder, denture stomatitis (Budtz Jorgensen 1974; Catalan *et al*. 1987; O'Donnell *et al*. 2015). Denture stomatitis is a common and problematic disorder for denture wearers (Hannah *et al*. 2017). Its progression is characterized by an inflammation and erythema of the oral mucosa in contact with the denture where it is in close proximity to denture plaque. Affecting between 15 and 70% of denture wearers (Gendreau and Loewy 2011), this condition causes discomfort and tissue swelling, often resulting in dentures becoming ill fitting and thus difficult to wear. In turn, this can result in loss of functionality and influence dietary and lifestyle choices that can dramatically affect quality of life for denture wearers. In addition to this, the onset and development of denture stomatitis can ultimately result in the denture having to be removed and replaced because treatments are often unsuccessful. This is a costly and demanding procedure for our health services (Polzer *et al*. 2010).

Over the years, the aetiology and management of denture stomatitis has been a particular focus of research, with attempts being made to clarify the causes of this condition—with *C. albicans* repeatedly being noted in this context. Many contributing factors have been implicated. Ramage *et al*. (2004) utilized scanning electron microscopy to visualise *in vivo* denture plaque biofilms from patients with denture stomatitis and noted a large amount of established yeast and hyphal cells, indicating an important role for *C. albicans* biofilms in denture stomatitis. In a large cohort study, Figueiral *et al*. (2007) investigated the causative factors in 124 denture‐wearing patients (54 with clinical symptoms of denture stomatitis, 70 without), showing that denture stomatitis was related to trauma caused by poorly fitting dentures, and could be increased by the long term and overnight wearing of dentures as well as denture age. This and other work supports previous findings that the condition is strongly related to poor denture hygiene and the high prevalence of *Candida* spp. on denture surfaces (Coco *et al*. 2008). In addition to these factors, denture stomatitis can adversely affect those with underlying illness or where disruption to natural defences occurs, for example, immunosuppressive therapies/diseases (HIV/AIDS), diabetes, and old age (Bartholomew *et al*. 1987; Webb *et al*. 1998; Pires *et al*. 2002). Indeed, attention has been drawn to the potential risks of candida oral carriage/infection with mechanical ventilation necessitated by COVID‐19 (Jerônimo *et al*. 2022).

An individual might possess the same set of dentures for many years (Wright 1994). Over this time, one would hope that regular visits for dental treatment were made, to ensure that oral tissues remain healthy and that denture fit is maintained. Loose‐fitting dentures can not only cause discomfort, they can introduce difficulties with eating (and hence nutrition) and speaking (hence socializing). Dentures that are poorly cared for can also be aesthetically unpleasant, being visibly dirty, or accompanying oral malodour. Little has been reported about denture malodour (Verran 2005), although the presence of oral anaerobes and/or yeast would likely be associated with the production of various volatile compounds. Denture‐associated odour has been noted as being ‘somewhat sweet, but unpleasant and readily identifiable’, yet studies on malodour tend to focus on the production of volatile sulphur compounds (Nalcaci and Baran 2008; Mousa *et al*. 2022). Like most other fungi, *C. albicans* and many bacterial species produce microbial volatile organic compounds (MVOCs), with over 250 described in the literature (Morath *et al*. 2012). Thus, is is important to consider the origin of the ‘sweet but offensive’ odour, and to characterize it more fully. Recent microbiome studies revealed a higher diversity of bacteria in patients with denture malodour, with bacteria including the phyla Fusobacteria and Firmicutes, and the genera Atopobium, Leptotrichia, Megasphaera, Oribacterium, and Campylobacter (Yitzhaki *et al*. 2018).

The denture has been identified as a reservoir of infection for a range of nonoral bacteria, some of which have been associated with inhalation pneumonia (O'Donnell *et al*. 2016; Takeuchi *et al*. 2019), and others which present resistance to a range of antibiotics (Lewis *et al*. 2015; Garbacz *et al*. 2019). Although it is possible that when dentures demonstrate colonization by micro‐organisms they may not be causing any negative consequence, it is important to ensure that good denture hygiene minimizes the likelihood of any serious infections that might arise from these organisms in the potentially immunodeficient elderly and those with systemic health disorders (Le Bars *et al*. 2015; Hannah *et al*. 2017; Jerônimo *et al*. 2022).

## Dental hygiene and treatment

As well as being essential to prevent the accumulation of denture plaque that may harbour potential pathogens, good denture hygiene is also required to limit malodour, and maintain good aesthetics in denture wearers (Jagger and Harrison 1995). There is a wide range of denture hygiene products and protocols available for denture cleaning (Ruiz Núñez *et al*. 2022). The UK National Health Service recommends that dentures are cleaned as often as normal teeth (i.e. every morning and night), brushing with either toothpaste or soap and water, supplemented by soaking in commercial cleansers on a regular basis and rinsing thoroughly (https://www.nhs.uk/conditions/dentures/). It is also recommended that dentures are removed at night. Improper cleaning regimes can be detrimental, for example, storing dentures in water might increase *Candida* colonization (Verhaeghe *et al*. 2020), and over‐abrasive dentifrices might damage the denture surface and impede cleaning (Verran *et al*. 2014), whilst one study found that the denture microbiome can remain relatively resilient to cleaning regimes (Delaney *et al*. 2019). However, if performed effectively and regularly, the recommended procedures should be adequate to maintain good denture hygiene. Indeed, in the first randomized double‐blinded control trial it was shown that frequent daily denture cleansing with a tablet and brushing was more effective than intermittent cleaning, significantly reducing microbial numbers in denture plaque (Ramage *et al*. 2019). Regular attendance at a dentist or hygienist would, therefore, benefit the average denture wearer in terms of oral health checks, including denture fit, hygiene monitoring, and evidence of yeast infection. Since denture hygiene would remove plaque and issues associated with plaque build‐up, one might anticipate reduced experience of denture‐associated infection. However, many denture wearers are elderly, and as a result often suffer from medical conditions such as arthritis and dementia, that can impair their ability to carry out these procedures effectively (Gornitsky *et al*. 2002), requiring assistance from carers and some education (Ruiz Núñez *et al*. 2022). Specific treatments are available if a *Candida* infection is suspected (Patil *et al*. 2015), with accompanying denture disinfection/cleaning or replacement (Garg and Garg 2010).

## Control and diagnosis of microbial colonization of dentures

One way to control denture colonization is to construct the denture to either prevent or reduce adhesion and attachment in the first instance, or retard growth and biofilm formation. The uneven nature of the denture‐fitting surface enhances cell retention and makes cleaning more difficult, but some elements of surface topography are essential to enhance fit. The relationship between the size of cell and surface feature (Whitehead *et al*. 2005; Sterzenbach *et al*. 2020) is key to engineering surface structures, such as the use of highly ordered nanopit topographies which have been shown to significantly reduce adherence capacity of *C. albicans* (Alalwan *et al*. 2018). On a note of caution, the dentifrice and brushing itself can cause abrasion to the denture, and enhance subsequent retention of micro‐organisms on the abraded surfaces (Verran *et al*. 2014).

**Figure 2:**
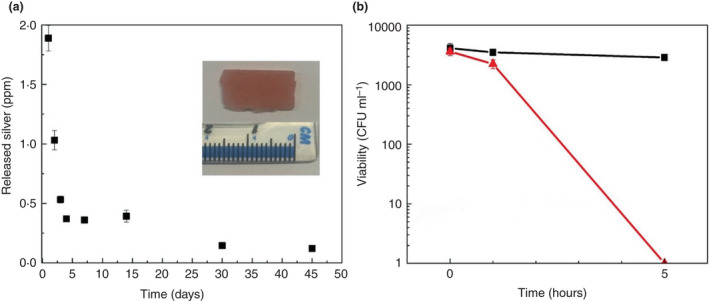
(a) Sustained silver release over 45 days from zeolite‐embedded denture acrylic treated with silver; digital image shown in the insert demonstrates preservation of resin’s aesthetic upon Ag loading into the zeolite‐embedded dental resin; and (b) Representative antimicrobial activity of the modified resin against a clinical strain of C. albicans after 5 hours having been incubated in distilled water for 45 days (Malic *et al*. 2019). Black square represents denture acrylic (polymethylmethacrylate) data, and red triangle represents denture acrylic embedded with zeolite and treated with silver. [Colour figure can be viewed at wileyonlinelibrary.com]

Inclusion of antimicrobial materials is another potential route to prevention of denture‐associated problems. Several recent reviews discuss different types of materials used, antimicrobial mechanisms, toxicity, and practical considerations (Allaker 2010; Gad *et al*. 2017; Imazato *et al*. 2020; Makvandi *et al*. 2020; Adam and Khan 2021; Garcia *et al*. 2021; Hao *et al*. 2021; Monteiro *et al*. 2021). Although a variety of materials has been suggested as potential antimicrobial additives in the literature, the studies are usually limited in the aspects of efficacy and performance assessment. In addition to antimicrobial and antibiofilm evaluation, there are essential criteria that denture materials must conform to, such as mechanical and aesthetic properties, cytotoxicity, leakage of substances from the dentures and their *in vivo* effect, antimicrobial lifetime, and so on. Examples of antimicrobial materials used as additives, their role and some critical considerations are provided in Table 2, and an example of zeolite‐embedded denture acrylic is provided in Fig. 2. As evident from Table 2, although a variety of materials having demonstrated their potential as filler materials to frabricate antimicrobial dentures, the lack of *in vivo* studies and clinical trials, in addition to shortcomings in the comprehensiveness of materials assessment, are the main barriers to their widespread practical application.

**Table 2:** Examples of filler materials for the fabrication of antimicrobial dentures

Material	Function/Notes	Critical considerations	Reference
Quaternary ammonium methacryloxy silicate (0.4, 2, 4, and 6 wt%)	Antimicrobial (*S. mutans*, *A. naeslundii*, *C. albicans*); sustained activity after 3 months water aging Antiadhesive (*C. albicans*) Clinical trial later reported (5 wt%)	Mechanical properties not studied; single‐species biofilms studied	Gong *et al*. (2013); Liu *et al*. (2016)
Silver nanoparticles (1, 2, 3, and 5 wt%)	Antibiofilm (*C. albicans*)	Mechanical and aesthetic properties not measured; single‐species biofilms studied; Ag release not determined; cytotoxicity not studied	Li *et al*. (2016)
Zeolite‐embedded silver ions (2 wt% zeolite; post‐synthesis loading of silver)	Antimicrobial (*C. albicans*, *S. mutans*, *F. nucleatum*), active for at least 45 days with possibility for silver recharging	Silver recharging possibility not verified; possible effects of long‐term silver exposure not studied; antibiofilm potential not studied	Malic *et al*. (2019)
Zinc oxide nanoparticles (2.5, 5 and 7.5 wt%)	Antifungal (*C. albicans*)	Antimicrobial lifetime not determined; long‐term zinc release not studied; flexural strength not measured	Cierech *et al*. (2016, 2018, 2019)
Carboxylated multiwalled carbon nanotubes (0.25, 0.5 and 1 wt%)	Antiadhesive (*S. aureus*, *S. mutans*, *C. albicans*)	*In vivo* biocompatibility tests needed; long‐term studies not performed; aesthetic properties not reported	Kim *et al*. (2019)
Graphene oxide nanosheets (nGO) (0.25, 0.5, 1, and 2 wt%)	Antiadhesive (*S. aureus*, *S. mutans*, *C. albicans*, *E. coli*). Sustained activity against *C. albicans* after incubation in artificial saliva for up to 28 days	Poor dispersion of nGO; *In vivo* biocompatibility tests needed. Activity not sustainable beyond 28 days; aesthetic properties not reported	Lee *et al*. (2018)
Graphene‐Ag nanoparticles (G‐AgNp) (1 and 2 wt%)	Antibacterial (*S. aureus*, *S. mutans*, *E. coli*)	Antibacterial lifetime not studied; uncertainty about the mechanism of antibacterial action; aesthetic properties not reported	Bacali *et al*. (2020)
Surface prereacted glass ionomer (5, 10, and 20 wt%)	Antibiofilm (*C. albicans*)	Mechanical properties not measured; increased surface roughness; single‐species biofilms studied; released of compounds determined after 24 h only; cytotoxicity not studied	Tsutsumi *et al*. (2016)
TiO_2_ nanoparticles (0.2, 0.4, 0.6, and 1 and 2.5 wt%); 3D printing	Antibacterial (*C. scotti*). 18 months clinical assessment of patient‐centred outcomes later reported (0.6 wt%)	Limited antimicrobial assessment; antimicrobial lifetime not studied	Totu *et al*. (2017); Cristache *et al*. (2020)
Mesoporous silica nanoparticles (MSNs) (0.5, 1, 2.5, and 5 wt%) for loading of amphotericin	Antiadhesive (*C. albicans* and S. *oralis*), active for 2 weeks	Increased surface roughness; biodegradation of MSNs decreases antimicrobial lifetime	Lee *et al*. (2016)
Nanodiamonds (0.5, 1, and 1.5 wt%)	Antiadhesive (*C. albicans*)	Colour changes observed; mechanical and cytotoxicity properties not studied	Fouda *et al*. (2019)

As noted above, *Candida* is known to produce MVOC (Scotter *et al*. 2005). Fungal MVOCs have a range of potential applications (Morath *et al*. 2012), and the identification of unique MVOC profiles has enabled seeing the differentiation between *Candida* species (Hertel *et al*. 2016), which, whilst a presence/absence of *Candida* may be all that is required for the denture wearer, access to *Candida* species information may be of clinical interest. This would enable monitoring for emerging species of interest, generation of overall prevalence data, consideration and administration of appropriate treatment (or prevention strategy), and overall knowledge of any implications for disease prevention. Thus, technology capable of identifying a unique pattern of MVOCs may be able to aid early detection of *Candida* denture colonization, as has been seen with other fungal species (Bingley *et al*. 2012), thus providing a rapid pathway towards prevention and early treatment. Moreover, if we are able to fully and accurately map the microbiome and metabolome of denture biofilms then it may be possible to accurately predict the onset of denture stomatitis and, therefore, instigate a chemotherapeutic strategy earlier.

## Conclusion

Although the literature focusing on denture plaque is significantly less than that on dental plaque, over the past 40 years, there has been consistent and increasing attention paid to this unique oral biofilm and its associated contribution to health and well‐being. The role of *C. albicans* in the aetiology of denture stomatitis is generally acknowledged, and sociological aspects of denture health are recognized. Efforts to speed diagnosis, prevent ill‐health, and improve quality of life for the elderly denture wearer continue apace.

## Author Contributions

Conceptualization (JV, JR), Methodology (JV, JR, LT, MB, GR), Formal Analysis (JV, JR, LT, SM, MB, GR); Writing—Original Draft Preparation (JV, JR, LT, SM, MB, GR); Writing—Review & Editing (JV, JR, LT, SM, MB, GR).

## Data Availability

Data sharing not applicable to this article as no datasets were generated or analysed during the current study.
